# Supplementary material for: Ultrahigh thermoelectricity obtained in classical BiSbTe alloy processed under super-gravity
Source: Nat Commun. 2025 Aug 16;16:7645. doi: 10.1038/s41467-025-62611-2 (PMC12357899; doi:10.1038/s41467-025-62611-2)
Supplement: Supplementary file 1 — Supplementary Information [file 41467_2025_62611_MOESM1_ESM.pdf]

Supplementary information

Ultrahigh thermoelectricity obtained in classical BiSbTe alloy processed under super-gravity

Min Zhou[1,‡,*], Haojian Su[1,2,‡], Jun Pei[3], Li Wang[4], Hualu Zhuang[5], Jing-Feng Li[5,*], Kun Song[6], Haoyang Hu[6], Jun Jiang[6,*], Qinghua Zhang[7], Jiangtao Li[8] & Laifeng Li[1,*]

[1]State Key Laboratory of Cryogenic Science and Technology, Technical Institute of Physics and Chemistry, Chinese Academy of Sciences; Beijing, 100190, China.

[2]Centre of Materials Science and Optoelectronics Engineering, University of Chinese Academy of Sciences; Beijing, 100049, China.

[3]Beijing Municipal Key Laboratory of New Energy Materials and Technologies, School of Materials Science and Engineering, University of Science and Technology Beijing; Beijing, 100083, China.

[4]School of Mechanical Engineering, Tianjin Sino-German University of Applied Science; Tianjin, 300350, China.

[5]State Key Laboratory of New Ceramic Materials, School of Materials Science and Engineering, Tsinghua University; Beijing, 100084, China.

[6]Ningbo Institute of Materials Technology and Engineering, Chinese Academy of Sciences; Ningbo, 315201, China.

[7]Institute of Physics, Chinese Academy of Sciences; Beijing, 100190, China.

[8]Suzhou Institute for Advanced Research, University of Science and Technology of China; Suzhou, 215123, China.

[‡]These authors contributed equally: Min Zhou, Haojian Su.

[*]Corresponding author. Email: mzhou@mail.ipc.ac.cn (M. Z.); jingfeng@mail.tsinghua.edu.cn (J.-F.L.); jjun@nimte.ac.cn (J.J.); lfli@mail.ipc.ac.cn (L.F.L.)

Table of contents

Effective mass modeling 5

Calculation of the thermal transport properties 6

Fig. S1 The real photos of the super-gravity re-melting setup. 9

Fig. S2 The flow chart of fabricating $(Bi,Sb)_2Te_3$ ingots by "chemical furnace". 10

Fig. S3 The relationship between the radius (R_B) and the rising velocity (V_B) of bubbles in melts under different gravity coefficient (G/g). 11

Fig. S4 The powder X-ray diffraction patterns of all the samples before and after SGF-RM. 12

Fig. S5 The thermal diffusivity coefficient (λ) of the $(Bi,Sb)_2Te_3$ samples before and after SGF-RM. 13

Fig. S6 The temperature dependence of heat capacity (C_p) for the $(Bi,Sb)_2Te_3$ alloy. 14

Fig. S7 Reproducibility of the thermoelectric properties for the Te-rich $Bi_{0.48}Sb_{1.52}Te_{3.03}$ alloys after SGF-RM. 15

Fig. S8 Reproducibility of the thermoelectric properties for more BST-1-R samples with the same fabrication process with BST-1 16

Fig. S9 Temperature dependence of (a) electrical conductivity, (b) Seebeck coefficient, (c) thermal conductivity and (d) zT values for the $(Bi,Sb)_2Te_3$ samples before and after SGF-RM in the in-plane direction. 17

Fig. S10. The optical test results. 18

Fig. S11 The charge carrier transport properties. 19

Fig. S12 The PALS spectra. 20

Fig. S14 TEM images of the BST-1 sample. 22

Fig. S15 The typical SEM images, chemical compositions and measured density at different locations (top, middle, bottom) along the gravity-field direction for BST and BST-1 samples. 23

Fig. S16 Schematic illustration of pore distribution supposing the pores are spherical and uniformly distributed. 24

Table S1. The density and viscosity of $(Bi,Sb)_2Te_3$ melts[85] 25

Table S2. The density, relative density, and porosities of the $(Bi,Sb)_2Te_3$ samples before and after SGF-RM. 26
Table S3. Parameters for the calculation of the thermal transport properties. 27
Table S4. The thermoelectric properties of n-type counterparts. 28
References 29

Effective mass modeling

As a kind of degenerate semiconductor, the electrical transport properties can be analyzed by the Single Parabolic Band (SPB) Model with acoustic phonon scattering[6,7,40]. The Seebeck coefficient can be expressed as:

$$\alpha = \frac{k_B}{e}\left(\frac{F_1^2(\eta)}{F_0(\eta)} - \eta\right) \tag{S1}$$

The Lorenz number (L) is given by:

$$L = \left(\frac{k_B}{e}\right)^2 \frac{3F_0F_2 - 4F_1^2}{F_0^2} \tag{S2}$$

The charge carrier concentration can be expressed as:

$$n_H = 4\pi \frac{(2k_BTm^*)^{3/2}}{h^3} F_{\frac{1}{2}}(\eta) \tag{S3}$$

The charge carrier mobility is directly correlated with the nondegenerate limits of drift mobility (μ_0), which can be expressed as:

$$\mu_H = \mu_0 \frac{F_{-\frac{1}{2}}(\eta)}{2F_0(\eta)} \tag{S4}$$

The weighted mobility is always defined as:

$$\mu_W = \mu_0 \left(\frac{m*}{m_0}\right)^{3/2} \tag{S5}$$

The $F_j(\eta)$ is Fermi integral, which can be expressed as:

$$F_j(\eta) = \int_0^\infty \frac{x^j}{1+e^{(x-\eta)}} dx \tag{S6}$$

The electrical conductivity can be expressed as:

$$\sigma = n_H\mu_H e = 2\pi\mu_0 e \frac{(2k_BTm^*)^{3/2}}{h^3} \frac{F_{\frac{1}{2}}(\eta)F_{-\frac{1}{2}}(\eta)}{F_0(\eta)} = 2\pi e \frac{(2k_BT)^{3/2}\ {m_0}^{3/2}\mu_W}{h^3} \frac{F_{\frac{1}{2}}(\eta)F_{-\frac{1}{2}}(\eta)}{F_0(\eta)} \tag{S7}$$

Then the power factor can be expressed as:

$$PF = \alpha^2\sigma = 2\pi\mu_0 k_B^2 \frac{(2k_BTm^*)^{3/2}}{h^3 e} \left(\frac{F_1^2(\eta)}{F_0(\eta)} - \eta\right)^2 \frac{F_{\frac{1}{2}}(\eta)F_{-\frac{1}{2}}(\eta)}{F_0(\eta)} \tag{S8}$$

Calculation of the thermal transport properties

The expression of the Debye-Callaway's model is shown as follows[7,74]:

$$\kappa_L = \frac{\kappa_B}{2\pi^2 \overline{v}} \left(\frac{2\pi \kappa_B T}{h}\right)^3 \int_0^{\theta_D / T} \tau(x) \frac{e^x x^4}{(e^x - 1)^2} dx \tag{S9}$$

where k_B is the Boltzmann constant, $\overline{v}$ is the average speed of sound, T is the absolute temperature, h is the reduced Planck constant, θ_D is the Debye temperature, $x = h\omega / k_B T$ is the reduced phonon energy, ω is the phonon angular frequency. The total relaxation time $\tau(x)$ can be calculated according to the Matthiessen's rule[6,74,75]:

$$\tau_{tot}^{-1} = \tau_U^{-1} + \tau_N^{-1} + \tau_{PD}^{-1} + \tau_{DS}^{-1} + \tau_P^{-1} \tag{S10}$$

In the Debye-Callaway's model, the integrand item is the spectral lattice thermal conductivity:

$$\kappa_s(x) = \frac{\kappa_B}{2\pi^2 \overline{v}} \left(\frac{2\pi \kappa_B T}{h}\right)^3 \tau_{tot}(x) \frac{e^x x^4}{(e^x - 1)^2} \tag{S11}$$

The frequency dependence of the spectral lattice thermal conductivity can be calculated using the following model:

$$\kappa_s(\omega) = \frac{\kappa_B}{2\pi^2 \overline{v}} \left(\frac{2\pi \kappa_B T}{h}\right)^3 \tau_{tot}(\omega) \frac{e^x x^4}{(e^x - 1)^2} \frac{\hbar}{\kappa_B T} \tag{S12}$$

The lattice thermal conductivity can be obtained from the integral of $\kappa_s(x)$ or $\kappa_s(\omega)$:

$$\kappa_L = \frac{\kappa_B}{2\pi^2 \overline{v}} \left(\frac{2\pi \kappa_B T}{h}\right)^3 \int_0^{\omega_D} \kappa_s(\omega) d\omega \tag{S13}$$

or

$$\kappa_L = \frac{\kappa_B}{2\pi^2 \overline{v}} \left(\frac{2\pi \kappa_B T}{h}\right)^3 \int_0^{\theta_D / T} \kappa_s(x) dx \tag{S14}$$

In the above equations, κ_L is the lattice thermal conductivity, k_B is the Boltzmann constant, $\overline{v}$ is the average speed of sound, T is the absolute temperature, h is the reduced Planck constant, $\hbar$ is the reduced Planck constant ($\hbar = h/2\pi$), θ_D is the Debye temperature, x is the reduced resonant frequency ($x = \hbar\omega / \kappa_B T$), ω is the phonon angular frequency, respectively.

The contribution of Umklapp process to the relaxation time can be expressed as[74,76]:

$$\tau_U^{-1}(\omega)=\frac{\hbar\gamma^2}{\bar{M}\bar{v}^2\theta_D}\omega^2 T\exp^{-\theta_D/3T} \tag{S15}$$

$$\omega=\frac{k_B Tx}{\hbar} \tag{S16}$$

where, $\bar{M}$ is the average atomic mass of $Bi_{0.5}Sb_{1.5}Te_3$, γ is the Gruneisen parameter, θ_D is the Debye temperature, respectively.

The contribution of Normal process to the relaxation time can be expressed as[74,76]:

$$\tau_N^{-1}(\omega)=\beta\tau_U^{-1}(\omega)=\beta\frac{\hbar\gamma^2}{\bar{M}\bar{v}^2\theta_D}\omega^2 T\exp^{-\theta_D/3T} \tag{S17}$$

where, β is the ratio between the normal process and Umklapp phonon scattering.

The point defect scattering in $Bi_{0.5}Sb_{1.5}Te_3$ mainly comes from the anti-site defects (disordered arrangement of Bi and Sb atoms in the equivalent position), which can be expressed as[74,77]:

$$\tau_{PD}^{-1}(\omega)=\frac{\bar{V}\omega^4}{4\pi\bar{v}^3}\Gamma \tag{S18}$$

where, Γ is the point defect scattering parameter.

The contribution of dislocation scattering is expressed as follows[7,77]:

$$\tau_{DS}^{-1}=\tau_{DC}^{-1}+\tau_{DE}^{-1} \tag{S19}$$

where, the contribution of dislocation core is:

$$\tau_{DC}^{-1}(\omega)=N_D\frac{\bar{V}^{4/3}}{\bar{v}^2}\omega^3 \tag{S20}$$

The contribution of dislocation strain is:

$$\tau_{DE}^{-1}(\omega)=0.06B_D^2N_D\gamma^2\omega\{\frac{1}{2}+\frac{1}{24}(\frac{1-2\upsilon}{1-\upsilon})^2[1+\sqrt{2}(\frac{v_L}{v_T})^2]^2\} \tag{S21}$$

where, B_D is the effective Burger's vector, N_D is the dislocation density, γ is the Grüneisen parameter, υ is the Poisson's ratio, v_L is the longitudinal phonon velocity and v_T is the transverse

phonon velocity, respectively. [Noting: In equation[35], "0.06" is a corrected value.]

The contribution of grain boundary scattering to the relaxation time can be expressed as[27]:

$$\tau_{DC}^{-1}(\omega) = N_D \frac{\overline{V}^{4/3}}{\overline{v}^2} \omega^3 \tag{S22}$$

where D is the average grain size, which is considered as 5 μm according to the SEM images (**Fig. S15**).

The contribution of micro-pores:

Supposing the micro-pores are spherical and uniformly distributed, and 1 cm^{-3} has N mirco-pores. Then each $1/N$ cm^{-3} would have one micro-pore at the middle, as illustrated by the single square shown in **Fig. S16**. Also, the volume of the square can be presented as $(L+d)^3$. So, $1/N=(L+d)^3$. Supposing the porosity is ε in the matrix, $N=6\varepsilon/\pi d^3$. The distance between two pores (L) is as follows:

$$L = \sqrt[3]{\frac{1}{N}} - d = \sqrt[3]{\frac{\pi d^3}{6\varepsilon}} - d = (\sqrt[3]{\frac{\pi}{6\varepsilon}} - 1) \bullet d \tag{S23}$$

$$\varepsilon = \frac{\rho_0 - \rho}{\rho_0} \bullet 100\% \tag{S24}$$

where, d is the size of pores, ε is the porosity, ρ_0 is the theoretical density, ρ is the measured density.

According to the effective medium theory (EMT)[6,78,79], the relaxation time associated with the pore interface scattering is as follows:

$$\tau_P^{-1} = \frac{\overline{v}}{(\sqrt[3]{\frac{\pi}{6\varepsilon}} - 1) \bullet d} \tag{S25}$$

All parameters involved above are given in **Table S3**.

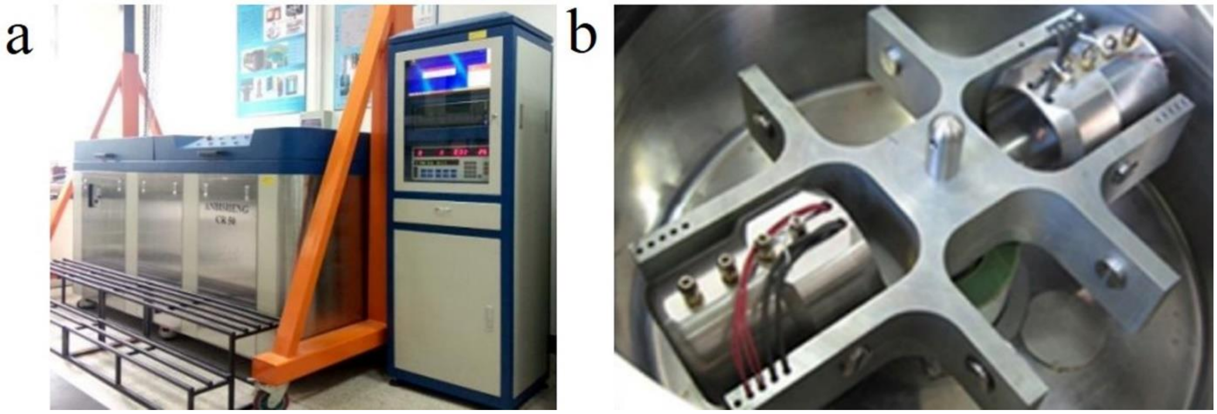

Fig. S1 The real photos of the super-gravity re-melting setup. (a) equipment appearance, (b) internal rotor.

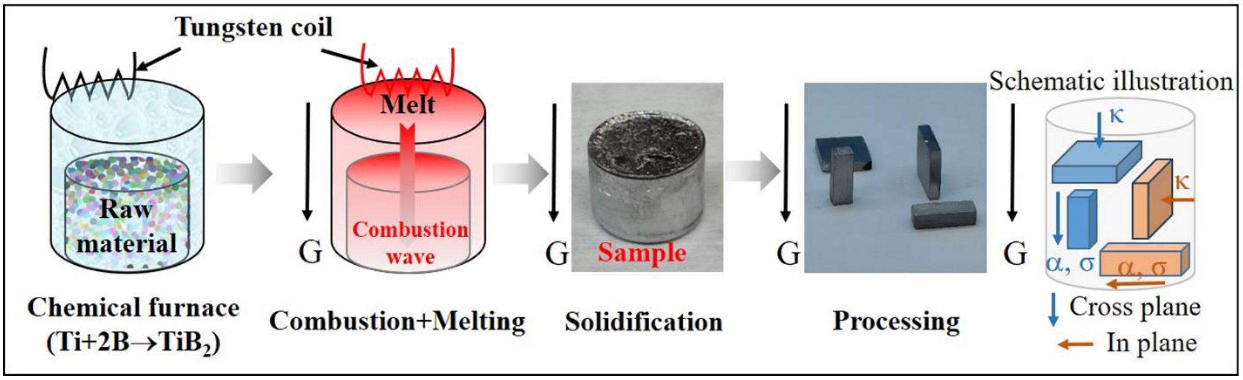

Fig. S2 The flow chart of fabricating $(Bi,Sb)_2Te_3$ ingots by "chemical furnace". The right two figures show the processed samples in different directions. Thermoelectric transport properties were also measured in different directions (cross-plane, in-plane directions).

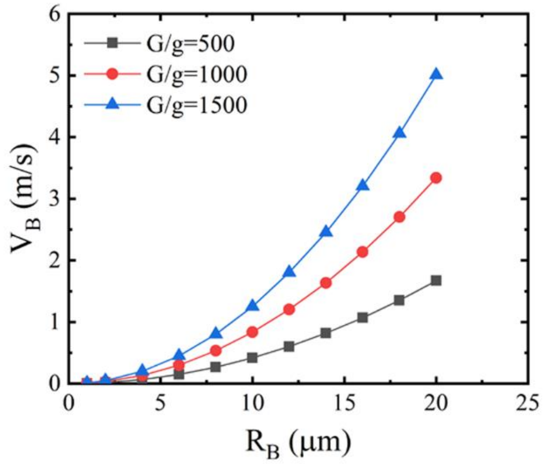

Fig. S3 The relationship between the radius (R_B) and the rising velocity (V_B) of bubbles in melts under different gravity coefficient (G/g).

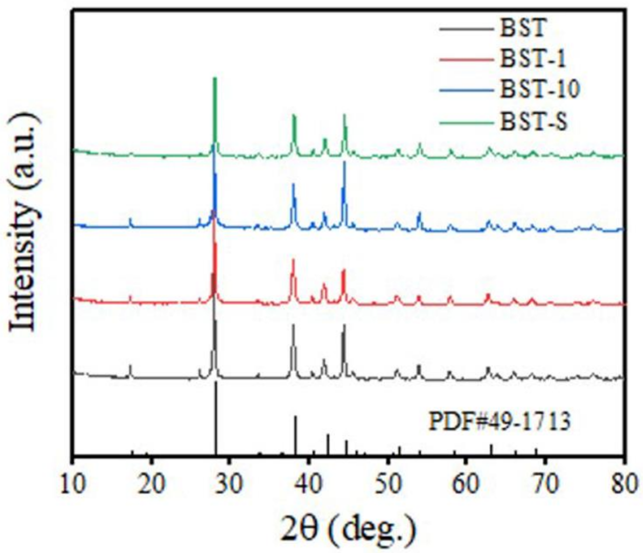

Fig. S4 The powder X-ray diffraction patterns of all the samples before and after SGF-RM.

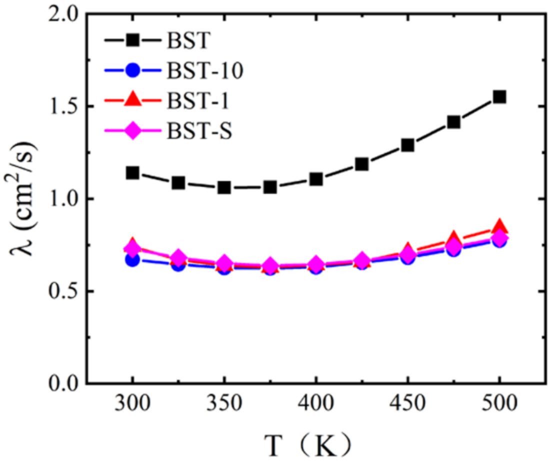

Fig. S5 The thermal diffusivity coefficient (λ) of the $(Bi,Sb)_2Te_3$ samples before and after SGF-RM.

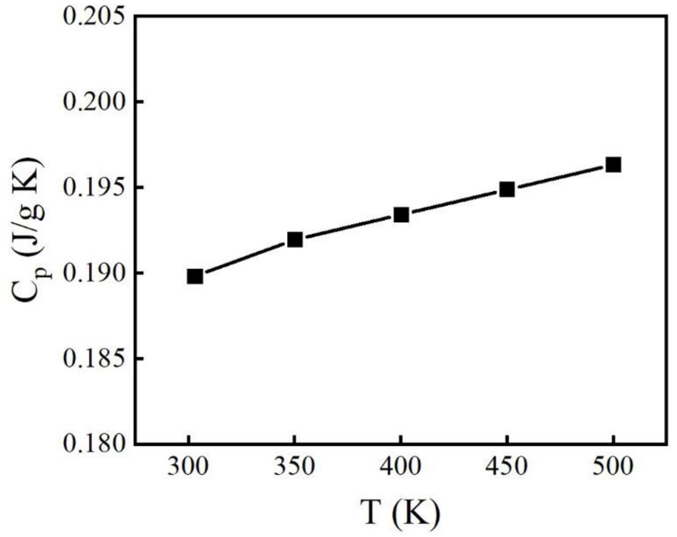

Fig. S6 The temperature dependence of heat capacity (C_p) for the $(Bi,Sb)_2Te_3$ alloy.

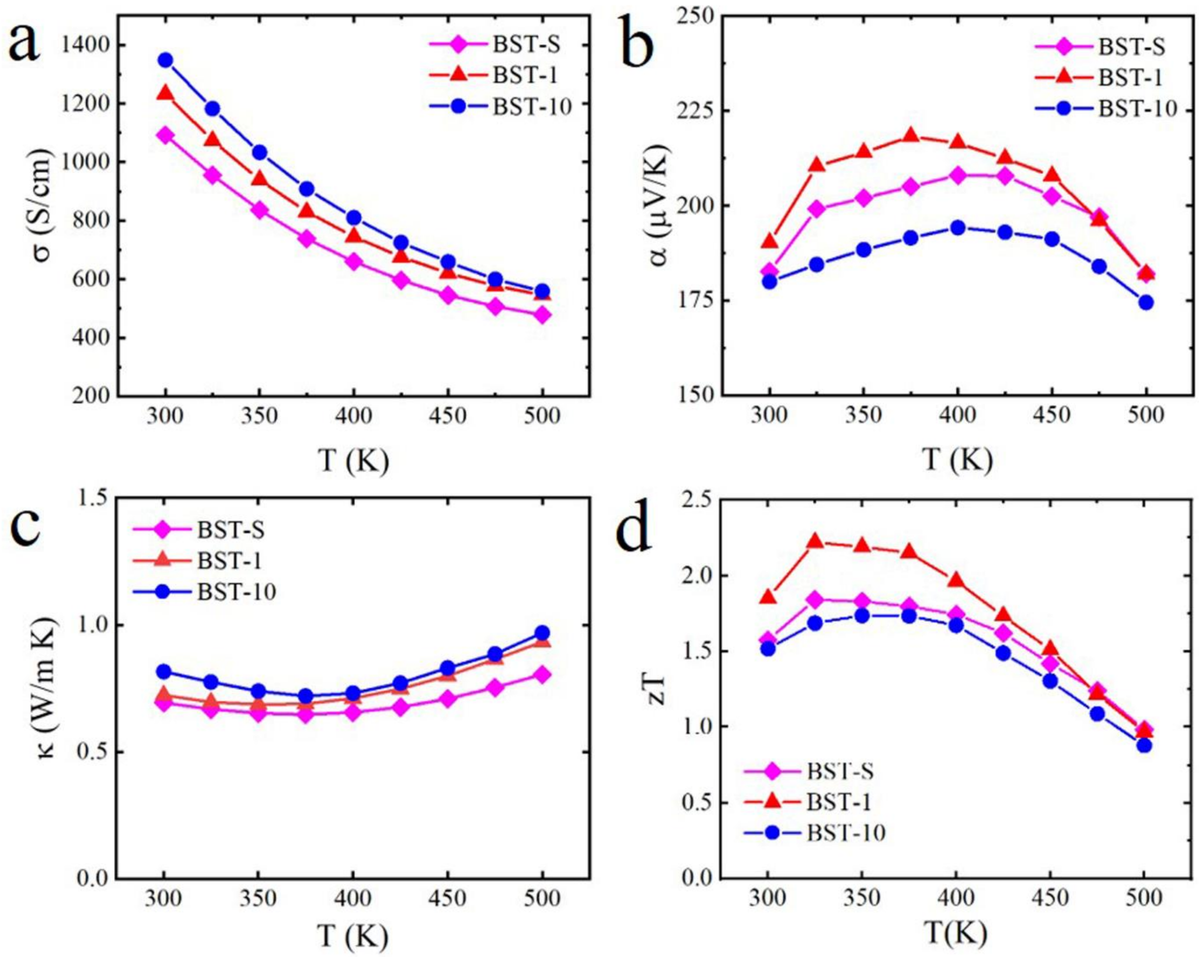

Fig. S7 Reproducibility of the thermoelectric properties for the Te-rich $Bi_{0.48}Sb_{1.52}Te_{3.03}$ alloys after SGF-RM. Temperature dependence of (a) electrical conductivity, (b) Seebeck coefficient, (c) thermal conductivity, (d) *zT* values. These repeated measurements were carried out in Beihang University.

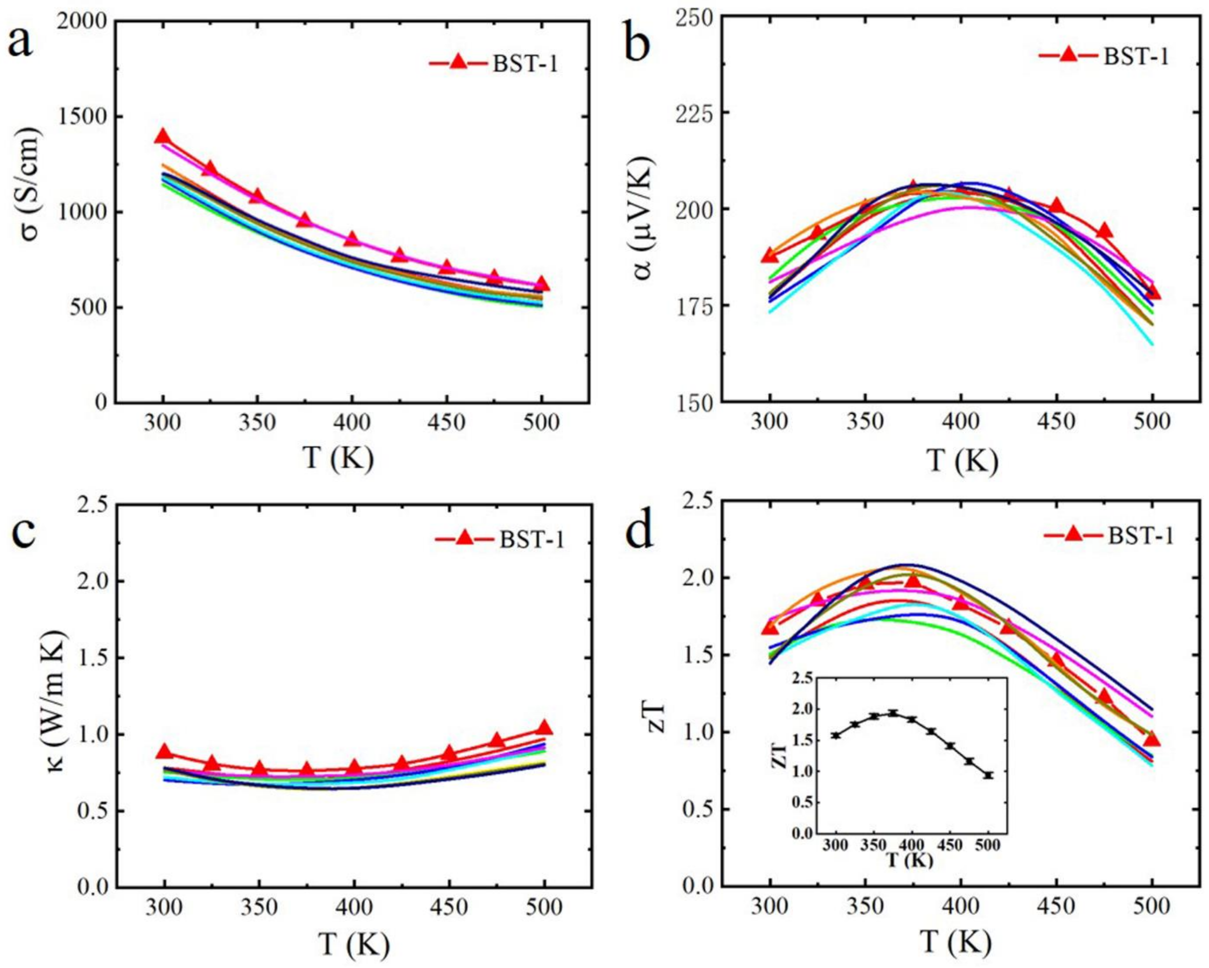

Fig. S8 Reproducibility of the thermoelectric properties for more BST-1-R samples with the same fabrication process with BST-1. Temperature dependence of (a) electrical conductivity, (b) Seebeck coefficient, (c) thermal conductivity, (d) *zT* values, inset image gives the average value of all the BST-1-R samples, which shows excellent reproducibility. The error bars represent the estimated standard error of *zT* values (<5%).

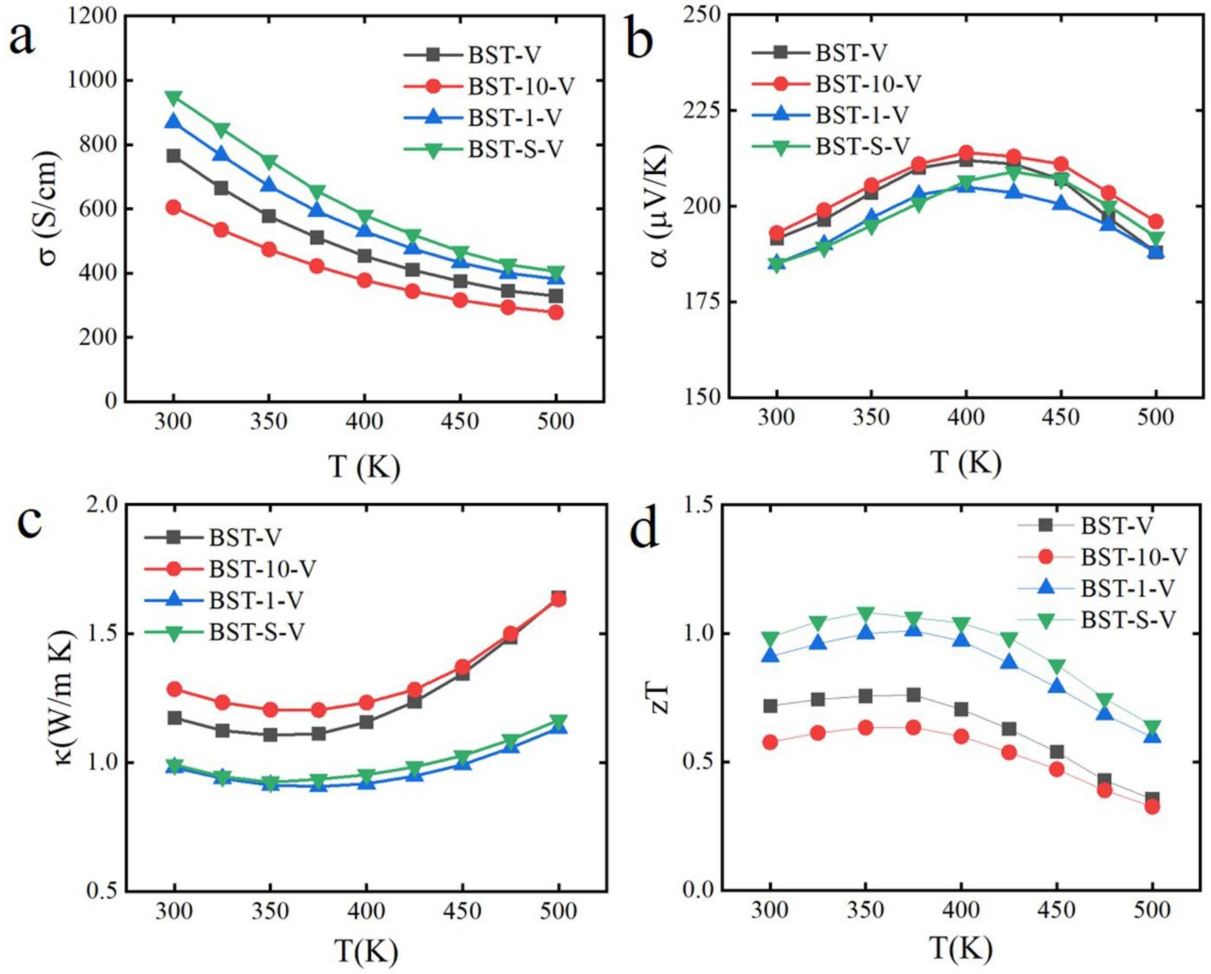

Fig. S9 Temperature dependence of (a) electrical conductivity, (b) Seebeck coefficient, (c) thermal conductivity and (d) zT values for the $(Bi,Sb)_2Te_3$ samples before and after SGF-RM in the in-plane direction.

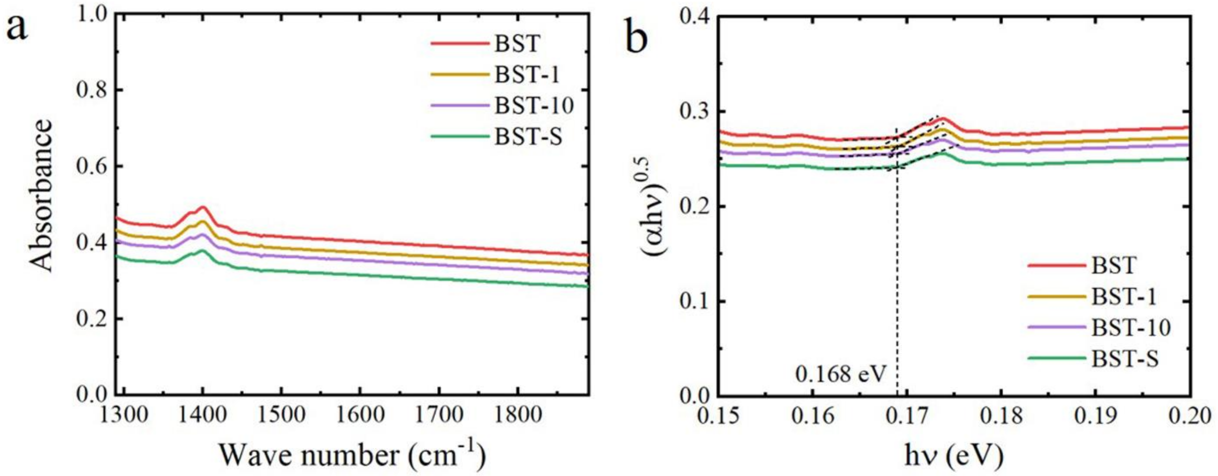

Fig. S10. The optical test results. (a) Fourier transform infrared absorption spectrum (middle-infrared band) of all samples, (b) Tauc plot of all samples. The dotted lines indicate the properly extrapolated technique. It is worth noting that the traditional method of directly extrapolating the linear segment of the Tauc plot for band gap semiconductor is inappropriate for use on degenerate semiconductors, where the occupation of conduction band energy states cannot be ignored[80, 81]. We used the properly extrapolated technique for extracting the optical band gap from absorption spectra by introducing a baseline function such that a vertical line (or ordinate) is then dropped to intersect the hν axis (Fig. S10b) [82,83]. The proper extrapolation band gap is about 0.168 eV for all the $(Bi,Sb)_2Te_3$ samples before and after SGF-RM. The reported band gap of $Bi_{0.5}Sb_{1.5}Te_3$ alloy is about 0.19 eV[84]. The estimated band gap values by the properly extrapolated technique are lower than the reported values. The small error in the band gap values may be related to the chemical compositions, grain sizes, porosity, fabrication process and extrapolation method etc.

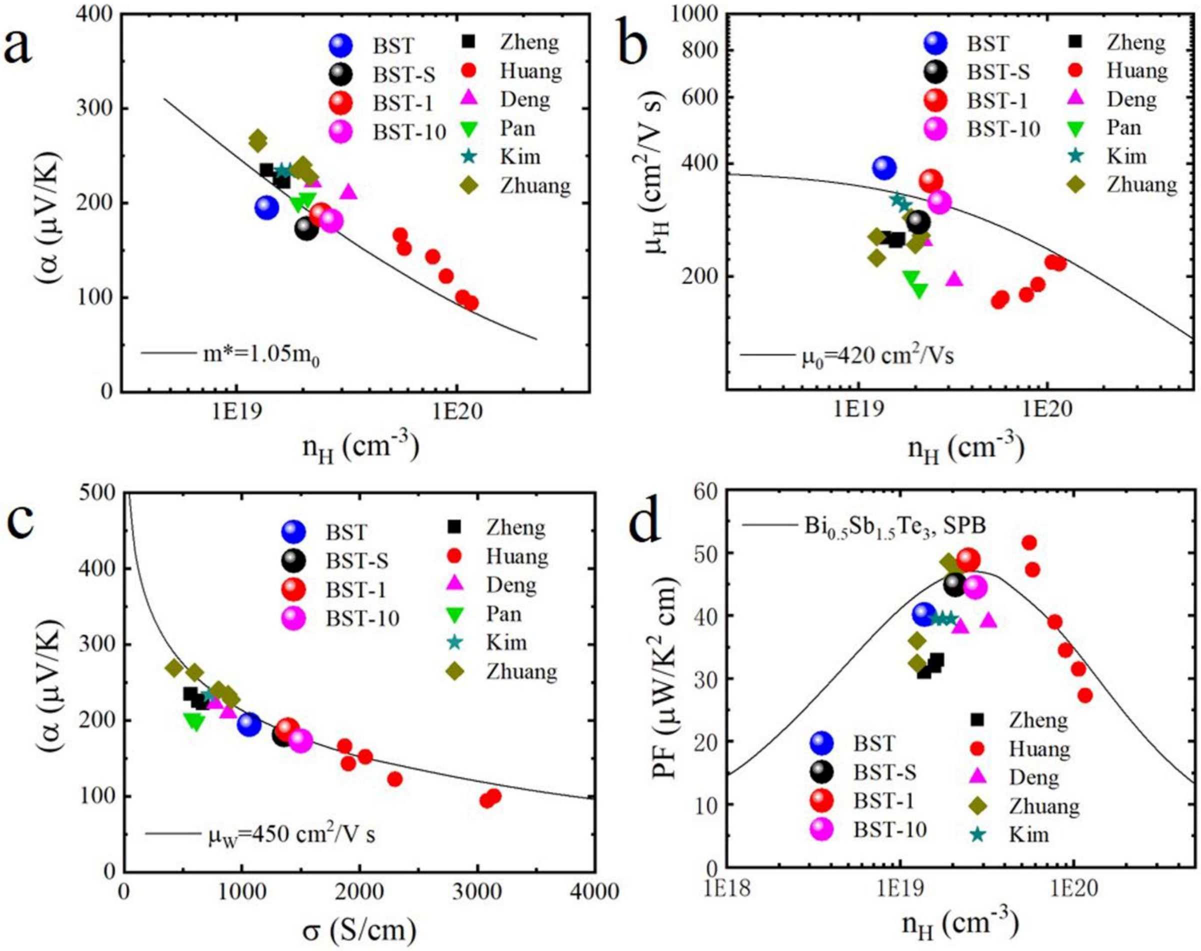

Fig. S11 The charge carrier transport properties. (a) Hall carrier concentration dependence of Seebeck coefficient (Pisarenko plot) at 300 K, (b) Hall carrier concentration dependence of Hall mobility at 300 K, (c) Electrical conductivity dependence of Seebeck coefficient at 300 K, (d) Power factor (PF) as a function of Hall carrier concentration predicted by $m^*=1.05\ m_0$ and $\mu_0=420\ cm^2/V\,s$ at 300 K. The bigger filled circles represent the data of this work. The other smaller filled symbols represent literature data[6,7,27,36,40,48].

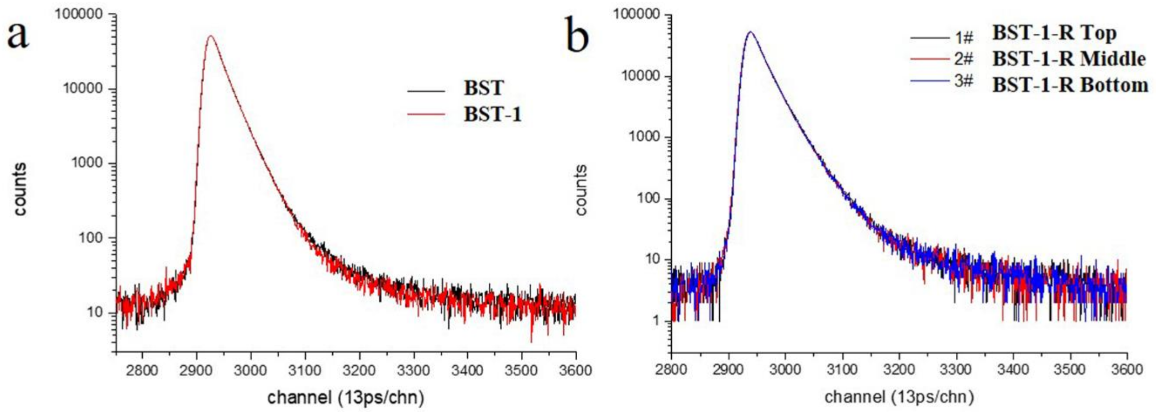

Fig. S12 The PALS spectra. (a) The PALS spectra of BST and BST-1 samples, (b) The PALS spectra at different locations (top, middle, bottom) along the gravity-field direction of BST-1-R sample.

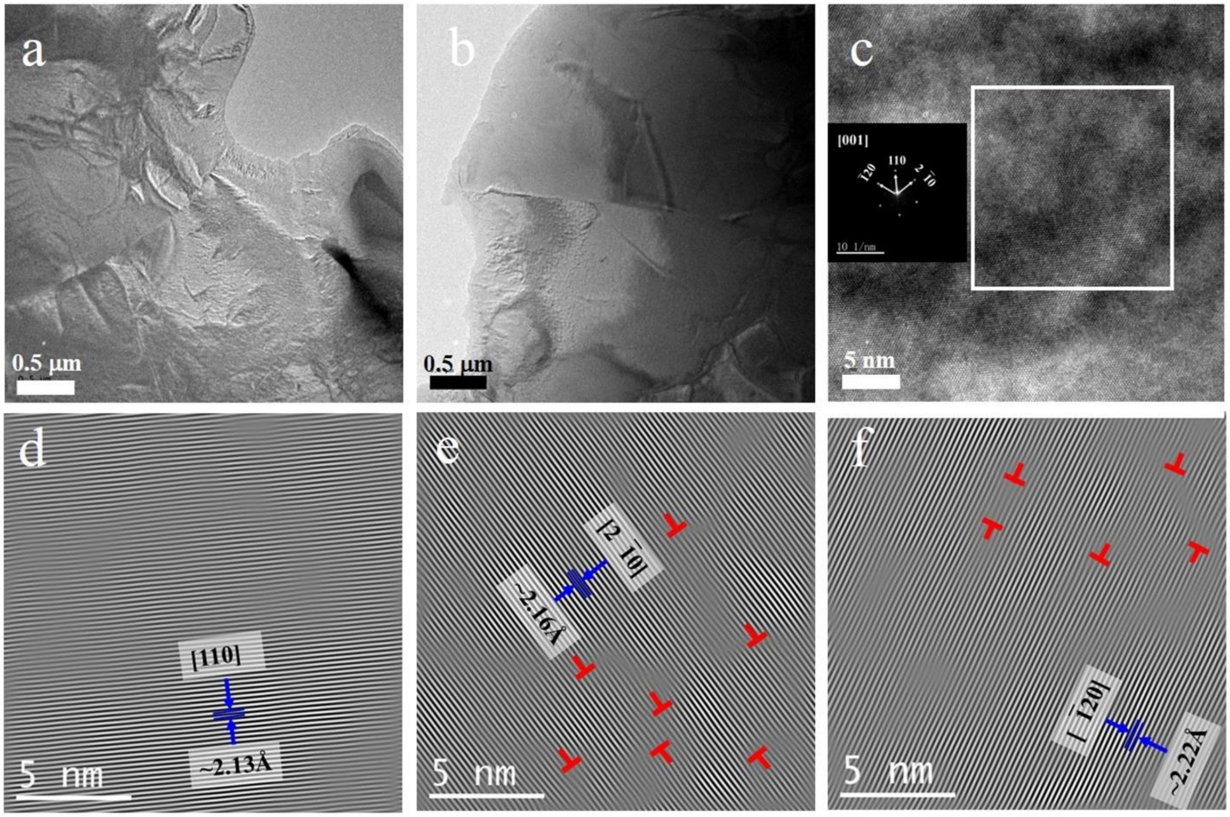

Fig. S13 TEM images of the BST sample. (a, b) Low-magnification in different regions, (c) high-magnification, (d–f) Inverse fast Fourier transform (IFFT) images in the (110), $(2\bar{1}0)$ and $(\bar{1}20)$ planes obtained from the area marked by the white rectangle in Fig. S12c.

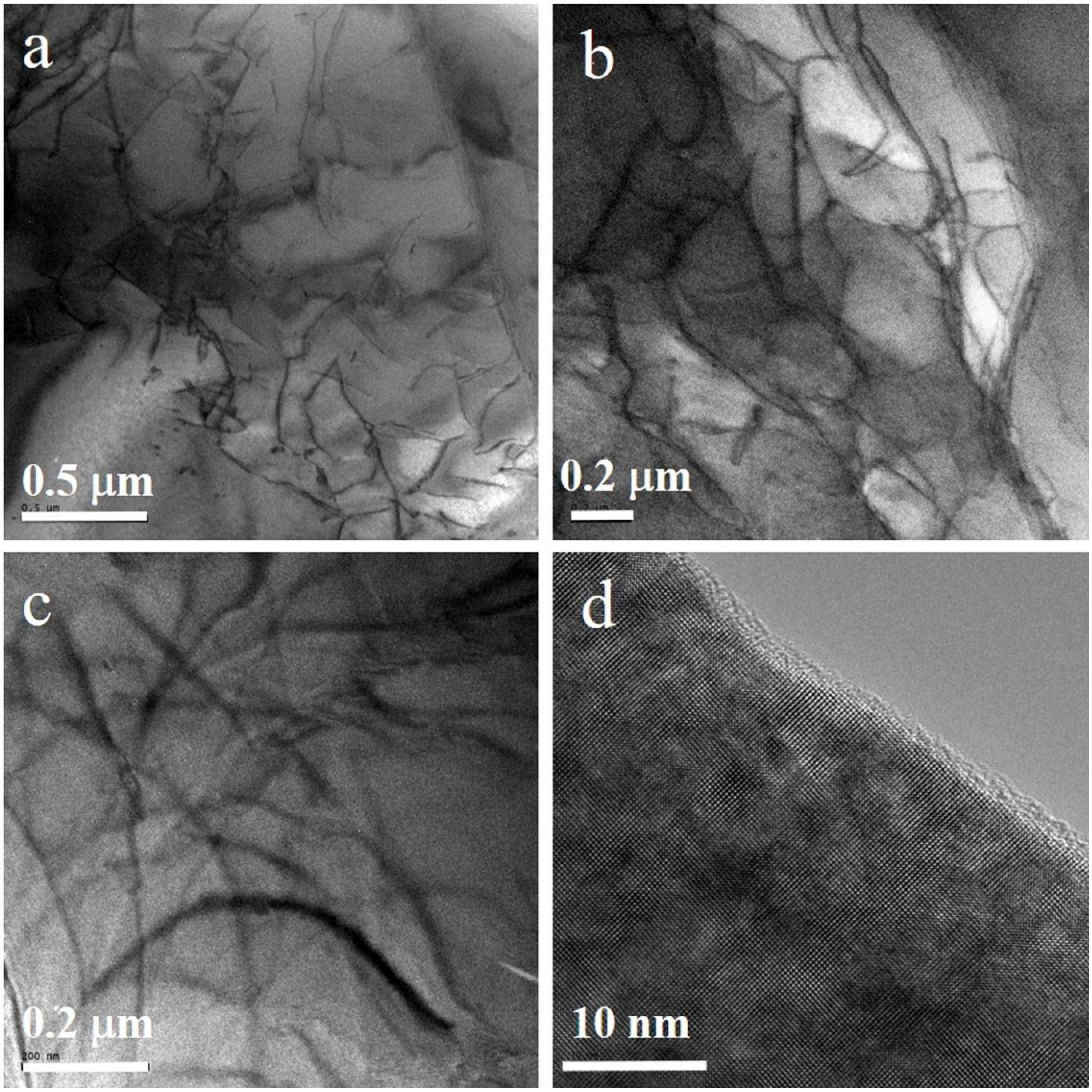

Fig. S14 TEM images of the BST-1 sample. (a, b, c) Low-magnification in different regions, (d) high-magnification.

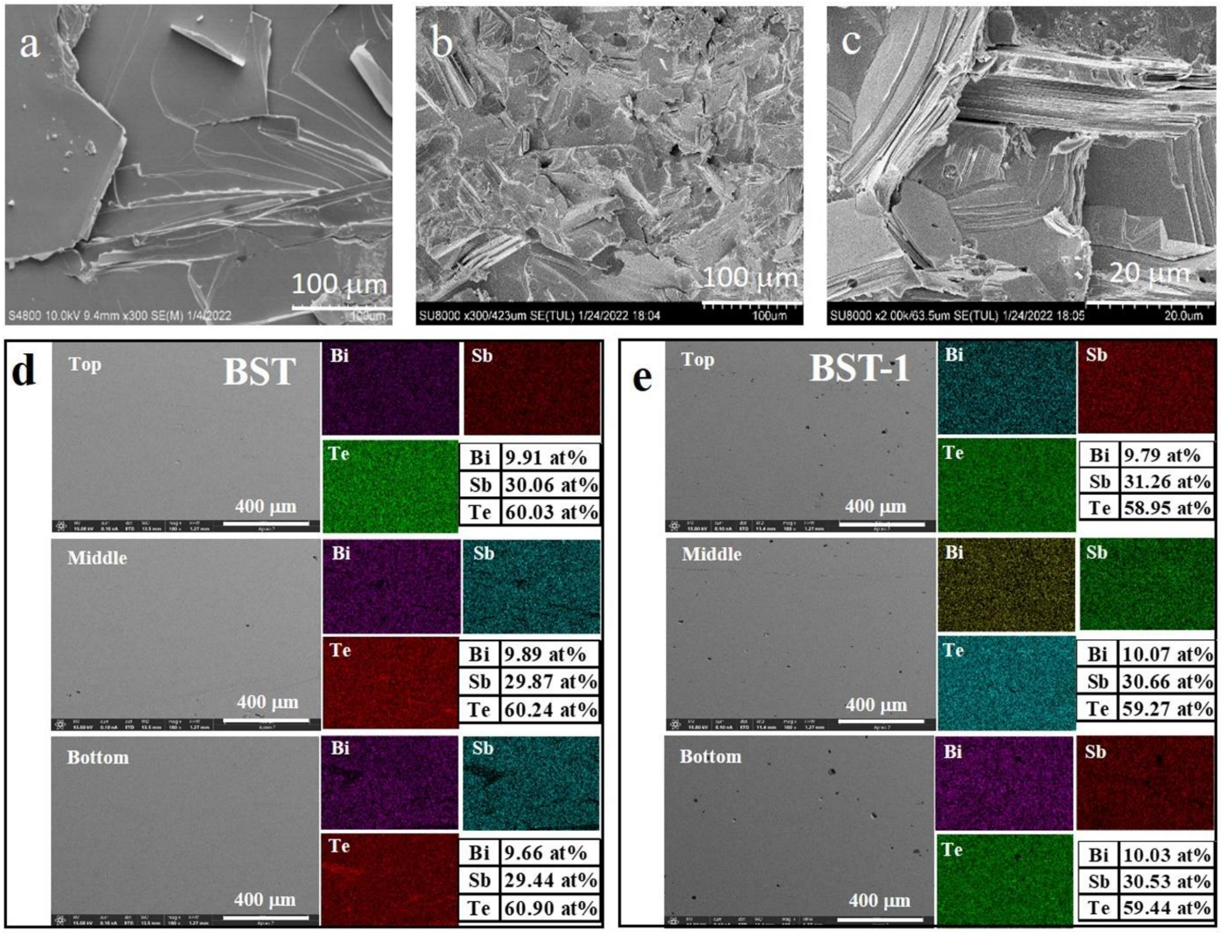

f

	BST			BST-1		
	Top	Middle	Bottom	Top	Middle	Bottom
Measured density	6.75	6.73	6.74	6.01	6.02	6.01

Fig. S15 The typical SEM images, chemical compositions and measured density at different locations (top, middle, bottom) along the gravity-field direction for BST and BST-1 samples. (a) The fractured surface SEM images of BST (lower magnification), (b) The fractured surface SEM images of BST-1 (lower magnification), (c) The fractured surface SEM images of BST-1 (higher magnification), (d) The polished surface SEM images and EDS results of BST, (e) The polished surface SEM images and EDS results of BST-1, (f) Measured density at different locations for BST and BST-1. The chemical compositions and density did not show obvious difference along the gravity-field direction.

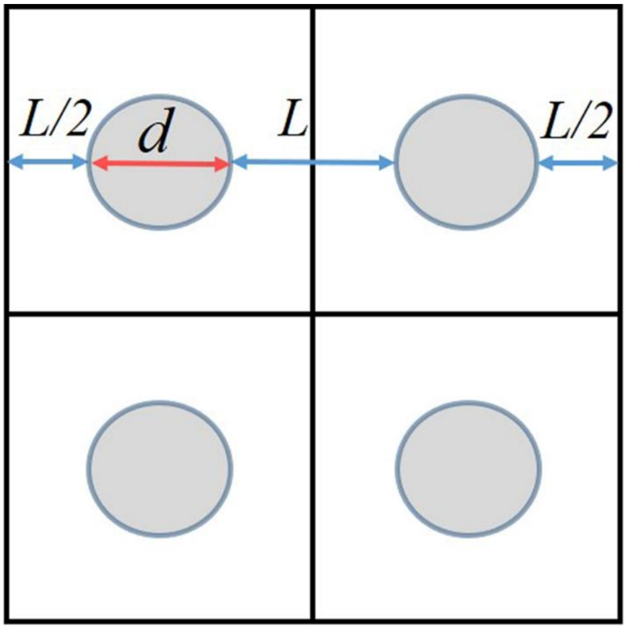

Fig. S16 Schematic illustration of pore distribution supposing the pores are spherical and uniformly distributed. Here shows four squares, each one has a volume of 1/N cm^{-3}.

Table S1. The density and viscosity of $(Bi,Sb)_2Te_3$ melts[85].

	Density (*g*/*cm*^{3})	Viscosity (*Pa S*)
Bi	10.03	0.0018
Sb	6.49	0.0012
Te	5.94	0.0020
$Bi_{0.48}Sb_{1.52}Te_{3.03}$	6.54	0.0017(calculated)

Table S2. The density, relative density, and porosities of the $(Bi,Sb)_2Te_3$ samples before and after SGF-RM.

samples	Measured density (g/cm^3)	Relative density (%)	Porosities $\Phi=100\%*(\rho_0-\rho)/\rho_0$	$(2-2\Phi)/(2+\Phi)$
BST	6.740	97.97	2.03	0.970
BST-1	6.016	87.44	12.56	0.823

Table S3. Parameters for the calculation of the thermal transport properties.

Parameters	Description	Values	Ref.
θ_D	Acoustic mode Debye temperature	94 K	(*86*)
$\bar{v}$	Cross-plane average speed of sound	2070 m/s (c-axis)	(*87*)
A	Comprehensive coefficient between Umklapp and Normal process	2.6	(*7*)
$\bar{V}$	Average atomic volume of $Bi_{0.5}Sb_{1.5}Te_3$	3.13×10^{-29} m^3	(*27*)
$\bar{M}$	Average atomic mass of $Bi_{0.5}Sb_{1.5}Te_3$	2.22×10^{-25} kg	Calculated
Γ	Point defect scattering parameter	0.145	(*27*)
B_D	Magnitude of Burgers vector	12.7 Å($12.7*10^{-10}m$)	(*27*)
N_D	Dislocation density of BST-1	7E12	Experimental
γ	Grüneisen parameter	2.3	(Sb_2Te_3)(*88*)
υ	Poisson's ratio	0.24	(*27*)
v_L	Longitudinal sound velocity along cross-plane	2539	(*87*)
v_T	Transverse sound velocity along cross-plane	1835	(*87*)
ρ_0	Theoretical density of the matrix	6.88 g/cm^3	(*25*)
ρ	Measured density of BST-1	6.016 g/cm^3	
ε	The porosity of BST-1	12.56%	
d	Pore average size of BST-1	1 μm	

Table S4. The thermoelectric properties of n-type counterparts.

Temperature (K)	Electrical conductivity (S/cm)	Seebeck coefficient (μV/K)	Thermal conductivity (W/m K)	*zT*
300	1000	-196	1.46	0.79
325	878	-214	1.43	0.91
350	790	-220	1.44	0.93
375	715	-223	1.5	0.89
400	663	-219	1.6	0.79
425	625	-213	1.71	0.7
450	608	-206	1.86	0.62
475	597	-195	2.02	0.53
500	600	-185	2.23	0.46

References

1. Bell, L. E. Cooling, heating, generating power, and recovering waste heat with thermoelectric systems. *Science* **321**, 1457–1461 (2008).
2. He, J. & Tritt, T. M. Advances in thermoelectric materials research: Looking back and moving forward. *Science* **357**, eaak9997 (2017).
3. Pei, Y. Z., Wang, H. & Snyder, G. J. Band Engineering of Thermoelectric Materials. *Adv. Mater.* **24**, 6125–6135 (2012).
4. Tan, G. J. et al. High Thermoelectric Performance of p-Type SnTe via a Synergistic Band Engineering and Nanostructuring Approach. *J. Am. Chem. Soc.* **136**,7006–7017 (2014).
5. Hu, L. P. et al. Tuning Multiscale Microstructures to Enhance Thermoelectric Performance of n-Type Bismuth-Telluride-Based Solid Solutions. *Adv. Energy. Mater.* **5**, 1500411 (2015).
6. Pan, Y. et al. Melt-Centrifuged $(Bi,Sb)_2Te_3$: Engineering Microstructure toward High Thermoelectric Efficiency. *Adv. Mater.* **30**, 1802016 (2018).
7. Zhuang, H. L. et al. Thermoelectric Performance Enhancement in BiSbTe Alloy by Microstructure Modulation via Cyclic Spark Plasma Sintering with Liquid Phase. *Adv. Funct. Mater.* **31**, 2009681 (2021).
8. Xie, W. J. et al. Unique nanostructures and enhanced thermoelectric performance of melt-spun BiSbTe alloys. *Appl. Phys. Lett.* **94**, 102111 (2009).
9. Lu, T. B. et al. Synergistically enhanced thermoelectric and mechanical performance of Bi_2Te_3 via industrial scalable hot extrusion method for cooling and power generation applications. *Mater. Today Phys.* **32**, 101035 (2023).
10. Goldsmid, H. J. Bismuth Telluride and Its Alloys as Materials for Thermoelectric Generation. *Materials* **7**, 2577–2592 (2014).
11. Witting, I. T. et al. The Thermoelectric Properties of Bismuth Telluride. *Adv. Electron. Mater.*

5, 201800904 (2019).

12. Kim, H.-S. et al. High thermoelectric performance in $(Bi_{0.25}Sb_{0.75})_2Te_3$ due to band convergence and improved by carrier concentration control. *Mater. Today* **20**, 452-459 (2017).
13. Jaworski, C. M., Kulbachinskii, V. & Heremans, J. P. Resonant level formed by tin in Bi_2Te_3 and the enhancement of room-temperature thermoelectric power. *Phys. Rev. B* **80**, 233201 (2009).
14. Heremans, J. P., Wiendlocha, B. & Chamoire, A. M. Resonant levels in bulk thermoelectric semiconductors. *Energ. Environ. Sci.* **5**, 5510-5530 (2012).
15. Lee, K. H. et al. Enhanced thermoelectric performance of n-type $Cu_{0.008}Bi_2Te_{2.7}Se_{0.3}$ by band engineering. *J. Mater. Chem. C* **3**, 10604-10609 (2015).
16. Kim, J. H. et al. Possible Rashba band splitting and thermoelectric properties in CuI-doped $Bi_2Te_{2.7}Se_{0.3}$ bulk crystals. *J. Alloy Compd.* **806**, 636-642 (2019).
17. Ishizaka, K. et al. Giant Rashba-type spin splitting in bulk BiTeI. *Nat. Mater.* **10**, 521-526 (2011).
18. Hu, L., Zhu, T., Liu, X. & Zhao, X. Point Defect Engineering of High-Performance Bismuth-Telluride-Based Thermoelectric Materials. *Adv. Funct. Mater.* **24**, 5211-5218 (2014).
19. Poudel, B. et al. High-thermoelectric performance of nanostructured bismuth antimony telluride bulk alloys. *Science* **320**, 634–638 (2008).
20. Scheele, M. N. et al. Synthesis and Thermoelectric Characterization of Bi_2Te_3 Nanoparticles. *Adv. Funct. Mater.* **19**, 3476–3483 (2009).
21. Shin, H. S. et al. Thermoelectric properties of 25%Bi_2Te_3-75%Sb_2Te_3 solid solution prepared by hot-pressing method. *J. Phys. Chem. Solids.* **58**, 671–678 (1997).
22. Lan, Y. C. et al. Structure Study of Bulk Nanograined Thermoelectric Bismuth Antimony Telluride. *Nano Lett.* **9**, 1419–1422 (2009).

23. Ma, Y. et al. Enhanced thermoelectric figure-of-merit in p-type nanostructured bismuth antimony tellurium alloys made from elemental chunks. *Nano Lett*. **8**, 2580–2584 (2008).

24. Tang, X. F. et al. Preparation and thermoelectric transport properties of high-performance p-type Bi_2Te_3 with layered nanostructure. *Appl*. *Phys*. *Lett*. **90**, 012102 (2007).

25. Shen, J. J. et al. Recrystallization induced in situ nanostructures in bulk bismuth antimony tellurides: a simple top down route and improved thermoelectric properties. *Energy Environ*. *Sci*. **3**, 1519–1523 (2010).

26. Cao, Y. Q. et al. Syntheses and thermoelectric properties of Bi_2Te_3/Sb_2Te_3 bulk nanocomposites with laminated nanostructure. *Appl*. *Phys*. *Lett*. **92**, 143106 (2008).

27. Kim, S. I. et al. Dense dislocation arrays embedded in grain boundaries for high-performance bulk thermoelectrics. *Science* **348**, 109–114 (2015).

28. Jo, S. et al. Simultaneous improvement in electrical and thermal properties of interface-engineered BiSbTe nanostructured thermoelectric materials. *J. Alloys Compd.* **689**, 899–907 (2016).

29. Deng, R. G. et al. Thermal conductivity in $Bi_{0.5}Sb_{1.5}Te_{3+x}$ and the role of dense dislocation arrays at grain boundaries, *Sci. Adv.* **4**, eaar5606 (2018).

30. Li, H. P. & Sekhar, J. A. Dimensional Changes during Micropyretic Synthesis. *Mat. Sci. Eng. A-Struct.* **160**, 221–227 (1993).

31. Paradis, P. F. & Ishikawa, T. Surface tension and viscosity measurements of liquid and undercooled alumina by containerless techniques. *Jpn. J. Appl. Phys. Part 1-Regular Papers Brief Communications & Review Papers* **44**, 5082–5085 (2005).

32. Glorieux, B. Millot, F. Rifflet, J. C. & Coutures, J. P. Density of superheated and undercooled liquid alumina by a contactless method. *Int. J. Thermophys.* **20**, 1085–1094 (1999).

33. Li, R. X. et al. Synthesis of $Al_xCoCrFeNi$ High-entropy Alloys by High-gravity Combustion

from Oxides, *Mat. Sci. Eng. A-Struct.***707**, 668–673 (2017).

34. Zhao, Z. M. et al. Microstructures and Properties of Large Bulk Solidified TiC-TiB_2 Composites Prepared by Combustion Synthesis under High Gravity, *Scripta Mater.* **61**, 281–284 (2009).
35. Zhao, Z. M. et al. Al_2O_3/ZrO_2 (Y_2O_3) Self-growing Composites Prepared by Combustion Synthesis under High Gravity, *Scripta Mater.* **58**, 207–210 (2008).
36. Liu, G. H. et al. Direct Fabrication of Highly-dense $Cu_2ZnSnSe_4$ Bulk Materials by Combustion Synthesis for Enhanced Thermoelectric Properties, *Mater. Design* **93**, 238–246 (2016).
37. Su, H. J et al. One-step Fabrication of a Bulk SnTe Thermoelectric Material with Excellent Performance through Self-propagating High-temperature Synthesis under a High-gravity Field, *Mater. Chem. Front.* **6**, 2175–2183 (2022).
38. Su, H. J et al. Fast Fabrication of SnTe via a Non-equilibrium Method and Enhanced Thermoelectric Properties by Medium-entropy Engineering, *J. Mater. Chem. C*, **11**, 5363–5370 (2023).
39. Khan, A. U. et al. Nano-micro-porous skutterudites with 100% enhancement in ZT for high performance thermoelectricity. *Nano Energy* **31**, 152–159 (2017).
40. Deng, R. G. et al. High thermoelectric performance in $Bi_{0.46}Sb_{1.54}Te_3$ nanostructured with ZnTe. *Energ Environ. Sci.* **11**, 1520–1535 (2018).
41. Li, C. C. et al. Magnetism-induced huge enhancement of the room-temperature thermoelectric and cooling performance of p-type BiSbTe alloys. *Energ Environ. Sci.* **13**, 535–544 (2020).
42. Mehta, R. J., Zhang, Y., Karthik, C. et al. A new class of doped nanobulk high-figure-of-merit thermoelectrics by scalable bottom-up assembly. *Nat. Mater.* **11**, 233–40 (2012).
43. Hyun, D. B. Hwang, J. S. You, B. C. Oh, T. S. & Hwang, C. W. Thermoelectric properties of

the n-type 85%Bi_2Te_3-15%Bi_2Se_3 alloys doped with SbI_3 and CuBr. *J. Mater. Sci.* **33**, 5595–5600 (1998).

44. Hwang, C. W. Hyun, D. B. Ha, H. P. & Oh, T. S. Effects of excess Te on the thermoelectric properties of p-type 25% Bi_2Te_3-75% Sb_2Te_3 single crystal and hot-pressed sinter. *J. Mater. Sci.* **36**, 3291–3297 (2001).
45. Li, F. et al. Enhanced thermoelectric performance of n-type bismuth-telluride-based alloys via In alloying and hot deformation for mid-temperature power generation. *J. Materiomics* **4**, 208–214 (2018).
46. Liu, W. et al. Studies on the Bi_2Te_3-Bi_2Se_3-Bi_2S_3 system for mid-temperature thermoelectric energy conversion. *Energ. Environ. Sci.* **6**, 552–560 (2013).
47. Cahill, D. G., Watson, S. K. & Pohl, R. O. Lower limit to the thermal-conductivity of disordered crystals. *Phys. Rev. B* **46**, 6131–6140 (1992).
48. Dong, J. et al. Reducing Lattice Thermal Conductivity of MnTe by Se Alloying toward High Thermoelectric Performance. *ACS Appl. Mater. Inter.* **11**, 28221-28227 (2019).
49. Zhang, C. C. et al. The Effect of Porosity and Milling Induced Defects on the Thermoelectric Properties of p-Type Bi_2Te_3-Based Bulks. *Adv. Eng. Mater.* **18**, 1777–1784 (2016).
50. Jiang, J. Chen, L. D. Bai, S. Q. Yao, Q. & Wang, Q. Thermoelectric properties of textured p-type $(Bi,Sb)_2Te_3$ fabricated by spark plasma sintering. *Scr. Mater.* **52**, 347–351 (2005).
51. Snyder, G. J. et al. Weighted Mobility. *Adv. Mater.* **32**, 2001537 (2020).
52. Zheng, Y. et al. Unraveling the Critical Role of Melt-Spinning Atmosphere in Enhancing the Thermoelectric Performance of p-Type $Bi_{0.52}Sb_{1.48}Te_3$ Alloys. *ACS Appl. Mater. Inter.* **12**, 36186–36195 (2020).
53. Huang, H. et al. Anisotropic thermoelectric transport properties of $Bi_{0.5}Sb_{1.5}Te_{2.96+x}$ zone melted ingots. *J. Solid State Chem.* **288**, 121433 (2020).

54. Dupasquier, A. & Mills Jr, A. *Positron spectroscopy of solids* (IOS press, 1995).

55. Jean, J. Y. Mallon, P. E. & Schrader, D. M. *Principles and applications of positron and positronium chemistry* (World Scientific, 2003).

56. Ning, X. et al. Modification of source contribution in PALS by simulation using Geant4 code. *Nuclear Instruments & Methods in Physics Research Section B-Beam Interactions with Materials and Atoms* **397**, 75–81 (2017).

57. He, H. F. et al. Interplay between Point Defects and Thermal Conductivity of Chemically Synthesized Bi_2Te_3 Nanocrystals Studied by Positron Annihilation. *J. Phys. Chem. C* **118**, 22389–22394 (2014).

58. Davoudi, K. M. & Vlassak, J. J. Dislocation evolution during plastic deformation: Equations vs. discrete dislocation dynamics study. *J. Appl. Phys.* **123**, 085302 (2018).

59. Messerschmidt, U. & Bartsch, M. Generation of dislocations during plastic deformation. *Mater. Chem. Phys.* **81**, 518–523 (2003).

60. Bennett, D. C. & Sawyer, B. Single crystals of exceptional perfection and uniformity by zone leveling. *Bell System Technical Journal* **35**, 637–660 (1956).

61. Hanus, R. et al. Lattice Softening Significantly Reduces Thermal Conductivity and Leads to High Thermoelectric Efficiency. *Adv. Mater.* **31**, 1900108 (2019).

62. Chen, Z. W. et al. Vacancy-induced dislocations within grains for high-performance PbSe thermoelectrics. *Nat. Commun.* **8**, 13828 (2017).

63. Wang, Y. et al. High Porosity in Nanostructured n-Type Bi_2Te_3 Obtaining Ultralow Lattice Thermal Conductivity. *ACS Appl. Mater. Inter.* **11**, 31237–31244 (2019).

64. Goldsmid H J. Introduction to Thermoelectricity. Berlin: Springer Press, 2010.

65. Hu, H. H. et al. Thermoelectric $Cu_{12}Sb_4S_{13}$-Based Synthetic Minerals with a Sublimation-Derived Porous Network. *Adv. Mater.* **33**, 2103633 (2021).

66. Zheng, G. et al. High thermoelectric performance of p-BiSbTe compounds prepared by ultra-fast thermally induced reaction. *Energ Environ. Sci.* **10**, 2638–2652 (2017).

67. Guo, Z. et al. Broadening the optimum thermoelectric power generation range of p-type sintered $Bi_{0.4}Sb_{1.6}Te_3$ by suppressing bipolar effect. *Chem. Eng. J.* **426**, 131853 (2021).

68. Hao, F. et al. High efficiency Bi_2Te_3-based materials and devices for thermoelectric power generation between 100 and 300 °C. *Energ Environ. Sci.* **9**, 3120–3127 (2016).

69. Wu, G. et al. Optimized Thermoelectric Properties of $Bi_{0.48}Sb_{1.52}Te_3$ through AgCuTe Doping for Low-Grade Heat Harvesting. *ACS Appl. Mater. Inter.* **13**, 57514–57520 (2021).

70. Bernert, T. et al. In situ observation of self-propagating high temperature syntheses of Ta_5Si_3, Ti_5Si_3 and TiB_2 by proton and X-ray radiography. *Solid State Sci.* **22**, 33–42 (2013).

71. Roy, S. K. et al. Combustion Synthesis of TiB and TiB_2 under vacuum. *J. Mater. Sci. Lett.* **13**, 371–373 (1994).

72. Rowe, D. W. et al. *CRC Handbook of Thermoelectrics* (CRC press, 1995).

73. Li, Z. Y. et al. Fine-Grained and Nanostructured $AgPb_mSbTe_{m+2}$ Alloys with High Thermoelectric Figure of Merit at Medium Temperature. *Adv. Energy Mater.* **4**, 1300937 (2014).

74. Hong, M. et al. Rashba Effect Maximizes Thermoelectric Performance of GeTe Derivatives. *Joule* **4**, 2030-2043 (2020).

75. Li, J. et al. Low-Symmetry Rhombohedral GeTe Thermoelectrics. *Joule* **2**, 976-987 (2018).

76. Toberer, E. S. Zevalkink, A. & Snyder, G. J. Phonon engineering through crystal chemistry. *J. Mater. Chem.* **21**, 15843-15852 (2011).

77. Zou, J. Kotchetkov, D. Balandin, A. A. Florescu, D. I. & Pollak, F. H. Thermal conductivity of GaN films: Effects of impurities and dislocations. *J.Appl. Phys.* **92**, 2534-2539 (2002).

78. Cohen, M. H. & Jortner, J. Effective Medium Theory for Hall-Effect in Disordered Materials.

Phys. Rev. Lett. **30**, 696-698 (1973).

79. Minnich, A. & Chen, G. Modified effective medium formulation for the thermal conductivity of nanocomposites. *Appl. Phys. Lett.* **91**, 073105 (2007).

80. Escobedo-Morales A. et al. Automated method for the determination of the band gap energy of pure and mixed powder samples using diffuse reflectance spectroscopy. *Heliyon* **5,** e01505 (2019).

81. Tauc J., Grigorovici R. &Vancu A., Optical properties and electronic structure of amorphous germanium, *Phys. Status Solidi* **15**, 627–637 (1966).

82. Jubu, P. R., Yam, F. K., Igba, V. M. & Beh, K. P. Tauc-plot scale and extrapolation effect on bandgap estimation from UV-vis-NIR data-A case study of β-Ga_2O_3. *J. Solid State Chem.* **290**, 121576 (2020).

83. Jubu, P. R. & Yam, F. K. Influence of growth duration and nitrogen-ambient on the morphological and structural properties of beta-gallium oxide micro- and nanostructures. *Mater. Chem. Phys.* **239**, 122043 (2020).

84. Sehr, R. & Testardi, L. R. Optical properties of p-type Bi_2Te_3-Sb_2Te_3 alloys between 2-15 microns. *J. Phys. Chem. Solids* **23**, 1219-1224 (1962).

85. Battezzati, L. & Greer, A. L. The Viscosity of Liquid-Metals and Alloys. *Acta Metallurgica* **37**, 1791-1802 (1989).

86. Bessas, D. et al. Lattice dynamics in Bi_2Te_3 and Sb_2Te_3: Te and Sb density of phonon states. *Phys. Rev. B* **86**, 224301 (2012).

87. Yang, F. Ikeda, T. Snyder, G. J. & Dames, C. Effective thermal conductivity of polycrystalline materials with randomly oriented superlattice grains. *J. Appl. Phys.* **108**, 0234310 (2010).

88. Chen, X. et al. Thermal expansion coefficients of Bi_2Se_3 and Sb_2Te_3 crystals from 10 K to 270 K. *Appl. Phys. Lett.* **99**, 261912 (2011).
